# PVDF Membrane-Based Dual-Channel Acoustic Sensor Integrating the Fabry–Pérot and Piezoelectric Effects

**DOI:** 10.3390/s23073444

**Published:** 2023-03-24

**Authors:** Qingkai Yao, Linfang Xie, Xing Guo, Fapeng Yu, Xian Zhao

**Affiliations:** Center for Optics Research and Engineering, State Key Laboratory of Crystal Materials, Shandong University, Jinan 250100, China

**Keywords:** PVDF, Fabry–Pérot, piezoelectric effect, convolution, resonance, signal-to-noise ratio

## Abstract

A resonant acoustic wave detector combined with Fabry–Pérot interference (FPI) and piezoelectric (PE) effects based on a polyvinylidene fluoride (PVDF) piezoelectric film was proposed to enhance the ability of the sensor to detect acoustic signals in a specific frequency band. The deformation of circular thin films was indicated by the interference and piezoelectric effects simultaneously, and the noise level was decreased by the real-time convolution of the two-way parallel signal. This study reveals that, at the film’s resonance frequency, the minimum detection limits for the FPI and piezoelectric impacts on acoustic waves are 3.39 μPa/Hz^1/2^ and 20.8 μPa/Hz^1/2^, respectively. The convolution result shows that the background noise was reduced by 98.81% concerning the piezoelectric signal, and by 85.21% concerning the FPI signal. The convolution’s signal-to-noise ratio (SNR) was several times greater than the other two signals at 10 mPa. Therefore, this resonance sensor, which the FPI and the piezoelectric effect synergistically enhance, can be applied to scenarios of acoustic wave detection in a specific frequency band and with ultrahigh sensitivity requirements.

## 1. Introduction

Presently, acoustic signal detection has critical applications in daily life, industrial production, medical health, and even in the national defense and military fields; for example, noise sensors [1] are used for large equipment and environmental noise acquisition and analysis; hydrophones [2] are used for seabed mapping, resource exploration, and the location of fish; health monitoring transducers [3,4] are used for the nondestructive structural inspection of composite materials and metal materials; and high-frequency ultrasonic imaging equipment is employed for the real-time monitoring of motion and nonionizing radiation in biomedical diagnosis [5]. The arrangement and application of acoustic sensors guarantee a high quality of production and life.

For piezoelectric sensors, traditional sensors are mainly bulk piezoelectric monocrystals and piezoelectric ceramics. These materials have a high piezoelectric coefficient and good temperature stability. However, they need to be more balanced and easier to process. Piezoelectric monocrystals benefit from good repeatability, stability, and multifunctionality, including electro–optical and acoustic–optical properties [6]. For instance, accelerometers, microbalances, oscillators, and other devices all employ quartz. In high-temperature acoustic sensors and transducers, crystals with high Curie temperatures, such as lithium niobate, are employed. Piezoelectric ceramics (PZT) are widely applied as transducer materials for sonar transducers and a wide range of ultrasonic applications in the MHz range [7]. Nonetheless, its comparatively high cost results from the difficulties in its manufacture. Compared to the crystals stated above, PVDF is primarily employed because of its high flexibility, coupled piezoelectric coefficient, and low weight [8]. Polymer piezoelectric materials have attracted the attention of researchers in recent years due to the fact of their outstanding performance, among which PVDF and its copolymers are the most widely used. This particular type of polymer material has a high reception sensitivity, as well as a high piezoelectric strain coefficient (d_33_) and piezoelectric voltage coefficient (g_33_) [9]. PVDF and its polymers can be prepared by casting, spin coating, inkjet printing, nanoimprinting, electrostatic spinning, and other methods. It has the characteristics of a low production cost and a short molding cycle. These molding processes can prepare polymer materials such as various special-shaped structures. For example, nano-sized films [10,11], microstructures [1], and nanofiber network structures [12,13] are suitable for layouts on curved surface structures [14] and different application scenarios.

The research and application of optical fiber as a sensor have a long history. With its long-distance transmission, low-loss optical transmission medium with electromagnetic interference resistance, corrosion resistance, good tensile strength, small mass, and relatively small size, it can reduce installation problems as much as possible. Many fiberoptic sensors now detect interference effects by producing different structures or using different materials. Examples include cascaded fiber Bragg grating (FBG), cascaded FPI, cascaded Mach–Zehnder interferometer (MZI), dual-channel SPR sensors, and any combination of various fiber structures [15,16,17,18,19]. Among them, nonintrinsic FPI sensors have been adopted to build high-sensitivity, wide band, and small-sized acoustic detection sensors using silver, silicon, and graphene films [20,21,22]. The FPI sensing head consists of two reflective surfaces: the tail-end face of the fiber and the reflective surface that generates displacement, forming a short F-P cavity. These sensors are advancing the field of static and dynamic pressure measurements [23]. The applications of high-quality graphene films are a current topic of great academic interest and research. Nevertheless, large-scale graphene is difficult to develop, which makes it difficult to commercialize [24].

Because of the low sensitivity, low detection limit, and weak noise suppression ability of nonresonant sensors in the field of weak signal detection, our research team proposed to adopt the first-order resonance frequency of the film as the acoustic detection frequency to improve the sensor’s sensitivity to the acoustic response and simultaneously monitor the piezoelectric and FPI signals of the diaphragm. Convolution was practiced to reduce the noise level and improve the signal-to-noise ratio. The resonant frequency is where the film frequency operates to make the sensor a high-performing one. Every object has a natural oscillation frequency, also called the resonant frequency. When an object is exposed to its natural frequency, the resonant frequency, it responds to that frequency and can even vibrate at the same frequency. In the development of the proposed resonant sensor, the frequency of the sound wave was consistent with the natural frequency of the film, which can cause the membrane to vibrate at the same frequency as the sound wave, improving the shape variable of the film and its ability to properly detect waves. In this paper, a PVDF piezoelectric film with a thickness of 40 μm and a diameter of 9 mm was applied as an acoustic–optical/acoustic–piezoelectric coupling diaphragm. The resonant frequency of the film was approximately 1570 Hz. The experimental results show that the lowest detection limits of the FPI effect and the piezoelectric effect on an acoustic wave are 3.39 μPa/Hz^1/2^ and 20.8 μPa/Hz^1/2^, respectively. Further, we conducted a convolution operation on the digital signals collected by the two channels. Regarding the convolution results, compared with the piezoelectric and FPI signals, the background noise levels decreased by 93.87% and 85.21%, respectively.

## 2. Design and Fabrication of Sensors

### 2.1. Design Principle

This paper’s manufactured sensor was fabricated in an extrinsic Fabry–Pérot interference (EFPI) working mode. The characteristics of the beam’s exit from the fiber are in general agreement with the Gaussian beam model. The silver film’s reflectance was approximately 0.995 when the incidence angle was near 0°. Unlike silicon, graphene, or other ultra-thin films, the boundary reflection properties can be calculated without using the Fresnel reflection theory. Since there is a divergence angle when the beam is emitted from the fiber end face, it is necessary to adjust the distance between the optical fiber and the PVDF membrane. Through the displacement of the adjustment platform for the modification of the silver film’s effective reflection coefficient, the maximum extinction ratio can be achieved. The cavity length (*L*) can be represented using FPI theory as [25,26,27]:

(1) $$L=\frac{\lambda_{1}\lambda_{2}}{2n(\lambda_{1}-\lambda_{2})},$$

where *λ*_1_ and *λ*_2_ are the wavelengths of the adjacent interference peaks in the interference fringe. The EFPI sensor’s interference intensity can therefore be described as

(2) $$I\left(\lambda \right)=I_{1}(\lambda )+I_{2}(\lambda )+2\sqrt{I_{1}{\left(\lambda \right)I}_{2}(\lambda )}\times cos(\frac{4\pi L}{\lambda }),$$

where *I*_1_(*λ*) is the fiber end face’s intensity, and *I*_2_(*λ*) is the silver film’s reflected intensity. It is possible to think of the silver film’s and the fiber face’s reflections as having a constant intensity. Therefore, it can be inferred that the change in the interference intensity caused by the change in the cavity length is the main factor. The expression of the change in the light intensity caused by the change in the cavity length caused by the vibration of the PVDF film can be expressed as

(3) $$∆I\left(\lambda \right)=-\frac{8\pi }{\lambda }\sqrt{I_{1}(\lambda )I_{2}(\lambda )}sin(\frac{4\pi L}{\lambda })∆L.$$

The differing light intensities are captured and demodulated to reveal the relationship between the acoustic wave and the signal.

When the acoustic frequency is consistent with the first-order resonant frequency of the circular diaphragm, the deformation of the diaphragm is much larger than that of the nonresonant frequency. In order to improve the sensitivity of the sensor, it is necessary to determine the resonant frequency of the diaphragm. The displacement of each diaphragm position at the resonance frequency must also be computed; arguably, this is even more crucial. Furthermore, the first-order resonance frequency (*f*_00_) can be expressed as follows using circular film resonance theory [28]:

(4) $$f_{00}=\frac{10.21t}{2\pi r^{2}}\sqrt{\frac{E}{12\rho (1-\nu^{2})}},$$

where *t*, *r*, *E*, rho, and *v* represent the film’s thickness (40 μm), radius (4.5 mm), Young’s modulus (5 × 10^9^ Pa), Poisson ratio (0.37), and mass density (1.82 × 10^3^ kg/m^3^), resulting in a resonant frequency of 1653 Hz.

**Figure 1:**
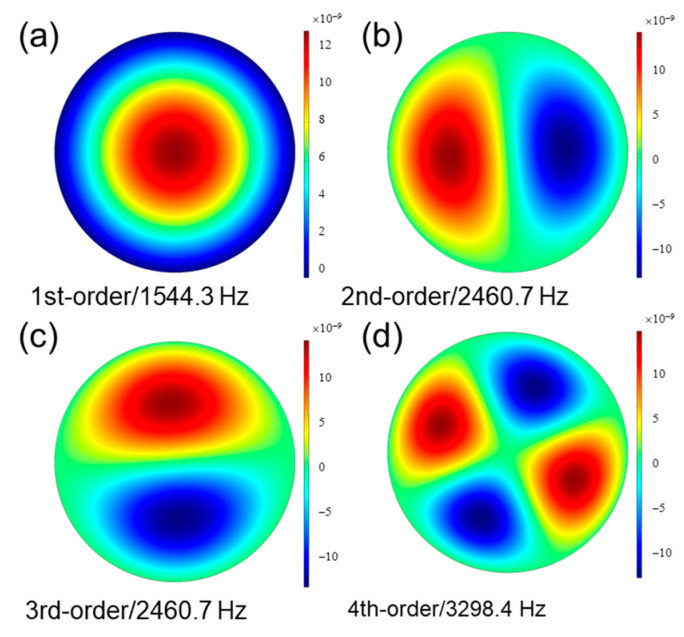
The simulation results of the first four-order resonant frequencies.

Employing a COMSOL multiphysical field simulation, a three-dimensional disk model of a 40 μm-thick PVDF piezoelectric film was modeled. The film’s resonant frequency was confirmed, and the right resonant frequency was chosen by calculating the distribution of the diaphragm displacement. The simulation results of the first four-order resonant frequencies and deflection distributions of the PVDF film with a diameter of 9 mm are shown in Figure 1. The second- and third-order deflection distributions are symmetrical and have two poles of maximum deformation. These two frequencies are not suitable for use as resonant frequencies for acoustic detection, because the deformation of the diaphragm center is small, which does not match the working mode of FPI for the detection of the deflection change characteristics of the film’s center. Similarly, the fourth-order resonant frequency with four extreme deformation points is also unsuitable. Therefore, we chose the first-order resonant frequency as the working frequency of the sensor to detect sound waves. The simulation results show that the first-order resonance frequency of the PVDF diaphragm with a diameter of 9 mm was approximately 1544 Hz. This frequency is consistent with the result calculated using Formula (4).

### 2.2. Fabrication of Sensor

In the experiment, a double-sided silver-coated PVDF film (Piezo Film, 1-1004346-0, Silver Ink, MEAS, Hampton, VA, USA) with a thickness of 40 μm was used, and the thickness of the silver film was approximately 12 μm. First, the PVDF film was cut into a circular sheet with a diameter of approximately 10 mm. Then, a rubber gasket with an inner diameter of 9 mm was used to clamp the piezoelectric film and install it into the designed housing to form the acoustic sensor. Next, the PVDF film and rubber gasket were fixed with nylon inserts and screws. A coaxial cable was used to connect the electrodes’ two ends onto the piezoelectric film. To ensure that the end face of the single-mode optical fiber was flat, it was cut with an optical fiber cutter. Then, the fiber was inserted into a cylindrical ceramic with a length of 10 mm, inner diameter of 125 μm, and outer diameter of 2.4 mm. The optical fiber and ceramic were fixed with epoxy resin, and the FPI cavity was formed with PVDF piezoelectric film. A high-precision, three-dimensional adjustment frame is required to create a sensor with high performance. The PVDF film and ceramic core that comprised the FPI cavity were controlled in terms of their length using the precision displacement platform. Variation in the length of the interference cavity causes the periodic variation of the interference intensity. The cavity length was adjusted to point Q by monitoring changes in the photodetector’s output to obtain the optimum electrical signal output value [22].

### 2.3. Composition of the Measurement System

**Figure 2:**
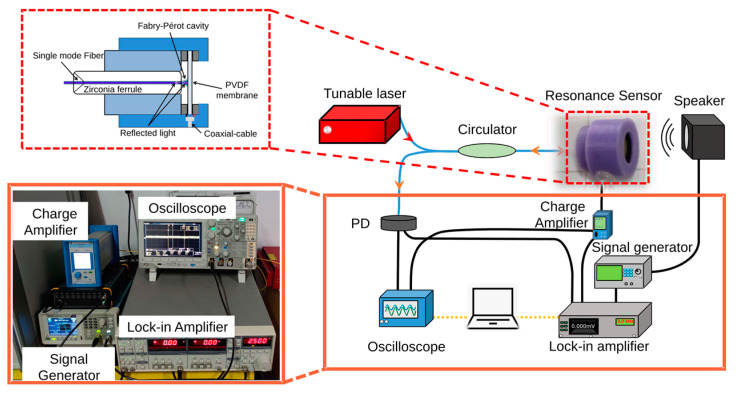
Schemes of the FPI and piezoelectric resonance acoustic detection system.

To eliminate interference from outside noise and acquire more precise acoustic data, the PVDF sensor was established in an acoustic isolation box. In this paper, a sound pressure meter (B&K 4189) was employed to calibrate the sound pressure level and obtain the sound pressure value in the sound insulation box in real time. The speaker was derived by a function signal generator (AFG1062, Tektronix, Beaverton, OR, USA) to produce acoustic signals of various frequencies and amplitudes. In addition, the resonant sensor and calibration sensor were situated on opposite sides of the symmetry to ensure the accuracy of the calibration. The light intensity changes produced by the Fabry–Pérot resonator formed by the optical fiber and film were transmitted to the photodetector (GDT-D002N, Daheng Optics, Beijing, China) by the fiber optic circulator. The charge generated by the piezoelectric effect was captured and amplified by a charge amplifier (5015A, KISTLER, Winterthur, Switzerland). The oscilloscope simultaneously captured the two signals, digitized them, and transmitted them to the upper computer. A schematic diagram of the system is shown in Figure 2.

## 3. Experimental Results and Discussion

### 3.1. Measurement of Resonant Frequency

The resonance frequency of the film is a crucial parameter, because this determines the sensor’s actual application scenario. A fundamental resonance frequency must be determined through a frequency response measurement. According to the results of the theoretical calculations and simulations, the frequency range of the test was selected as 500–6000 Hz, and the corresponding piezoelectric and interference outputs of the sensor were tested simultaneously. In the resonance mode, the film was susceptible to the sound wave, resulting in a significant shift in the center of the film. This caused the FPI effect to fluctuate over the period, resulting in signal distortion. Therefore, the FPI output characteristics were measured at a sound pressure of 50 mPa.

**Figure 3:**
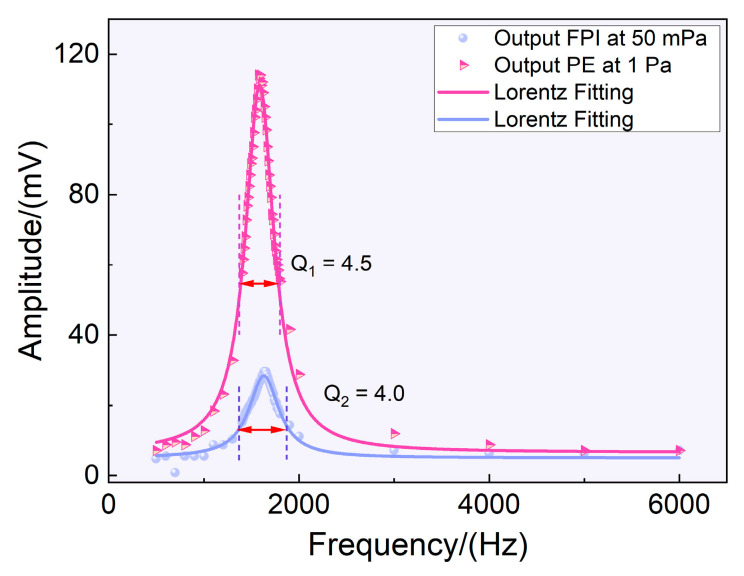
The formant of the FPI and piezoelectric effects.

As shown in Figure 3, the resonant peaks generated by the piezoelectric effect of the sensor and the FPI effect were consistent, both appearing at approximately 1570 Hz, which is roughly consistent with the theoretical value and simulation value of the first-order resonance frequency of the film. In addition, the quality factor of the resonance sensor was calculated. The quality factor, or *Q* factor, is a dimensionless parameter in physics and engineering representing the ratio of the energy stored to the energy dissipated in a system. The higher the *Q* value, the lower the system’s dissipation proportion. Therefore, the longer the average life of the energy storage, the more sensitive the sensor to sound waves at a central frequency. The results suggest that the two effects’ quality factors were identical.

*Q* = *f*/∆*f*. (5)

### 3.2. Sensor Performance

**Figure 4:**
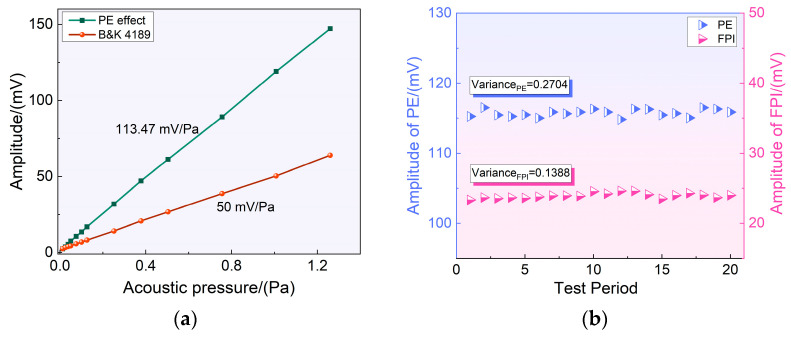
(**a**) Relationship between the piezoelectric effect signal and sound pressure level at the formant; (**b**) stability test of the resonance sensor.

The investigated sensor was operated at the formant peak to verify the linear response characteristics, and the output characteristics of FPI at different sound pressure levels were analyzed. The time-domain signal was acquired and filtered using LabVIEW software. After eliminating the DC component, the relationship between the peak–peak value of the piezoelectric effect signal at the formant frequency and sound pressure could be observed, as shown in Figure 4a. As can be seen, the commercial acoustic field calibration sensor had a sensitivity of 50 mV/Pa, while the piezoelectric effect had a sensitivity of 113.47 mV/Pa. In addition, the sensor’s stability was analyzed within 20 cycles, and as Figure 4b shows, the variance of the signal amplitude of both the FPI and PE can be ignored.

**Figure 5:**
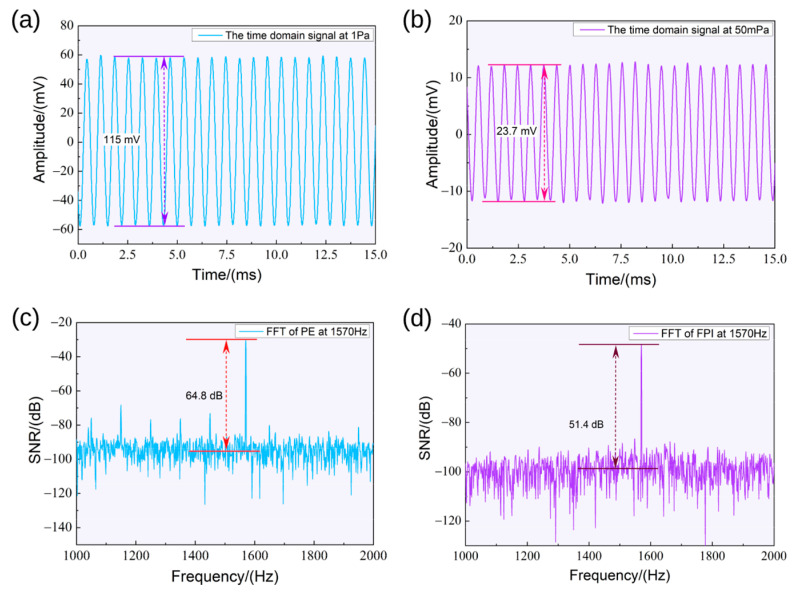
(**a**,**b**) Time-domain signals of the piezoelectric and FPI effects; (**c**,**d**) frequency-domain signals of the piezoelectric and FPI effects.

In addition, the time-domain signals of the piezoelectric effect under a 1 Pa sound pressure level and the FPI effect under 50 mPa were collected. As shown in Figure 5a,b, the time-domain signals were consistent with the sinusoidal excitation signals with no noticeable phase delay. This shows that the resonant sensor at the formant could better characterize the characteristics of the acoustic signals. The Fourier transform of the original signal, as depicted in Figure 5c,d, shows that the noise level was at approximately −100 dB. In addition, some peaks of nonexciting frequency components appeared, especially in the piezoelectric signals. This phenomenon occurs due to the operation of high-power electrical equipment in the surrounding area and the widespread use of mobile communication, wireless networks, and other similar technologies, which has also significantly increased sources of electromagnetic harassment.

### 3.3. Detection Limits

**Figure 6:**
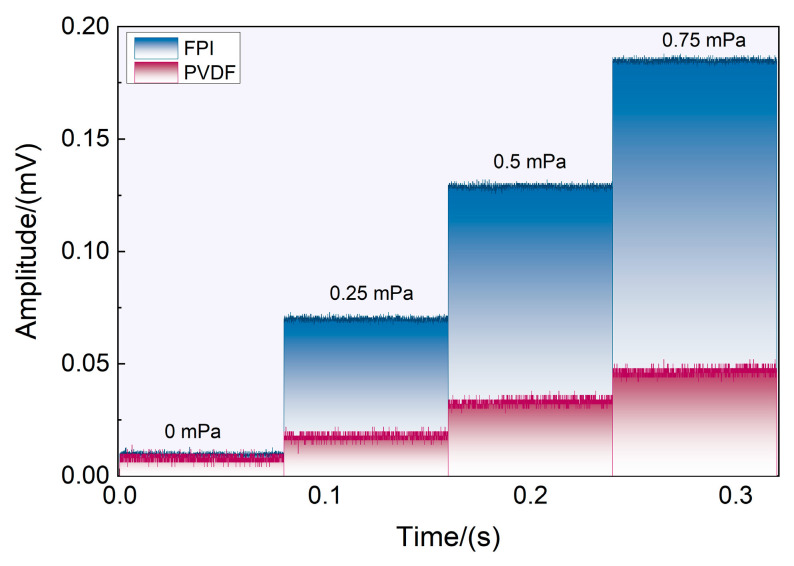
The output of the lock amplifier of the piezoelectric and FPI effects at different sound pressure levels.

In addition, advanced signal detection technology is effective at improving the ability of sensors to detect weak signals. For example, phase-locked technology has become a widely adopted weak signal detection technique in the laboratory. Therefore, the acoustic wave detection limit was detected in this paper utilizing a phase-locked amplifier (SR830, Stanford Research Systems, Sunnyvale, CA, USA) at the formants. The voltage values obtained by the phase-locked amplifier under different sound pressure levels obtained at the reference frequency of 1570 Hz are shown in Figure 5. The integration time of the phase-locked amplifier was set at 100 ms, and the slope was 18 dB/oct. The system’s output response was evaluated concurrently while subjected to background noise, as illustrated in Figure 6. Under 0.25 mPa, the output amplitudes were 0.0690 mV and 0.0178 mV. The detection limit can be obtained according to the following formula for the detection limit when combined with the noise level:

(6) $$P_{limit}=\frac{P}{\left((V-V_{0})/N_{\sigma }\right)},$$

where P denotes the sound pressure level, V denotes the lock-in amplifier’s signal amplitude, V_0_ is the signal output under silent wave stimulation, and Nσ denotes the noise amplitude. The lowest detection limits for the piezoelectric and FPI signals were 3.39 μPa/Hz^1/2^ and 20.8 μPa/Hz^1/2^. The lowest detection limit of the reference sensor B&K 4189 was approximately 8 μPa/Hz^1/2^. It is essential to point out that the lower detection limit can be obtained by further prolonging the integration time of the phase-locked amplifier.

### 3.4. Convolution Noise Reduction in Two-Channel Signals

**Figure 7:**
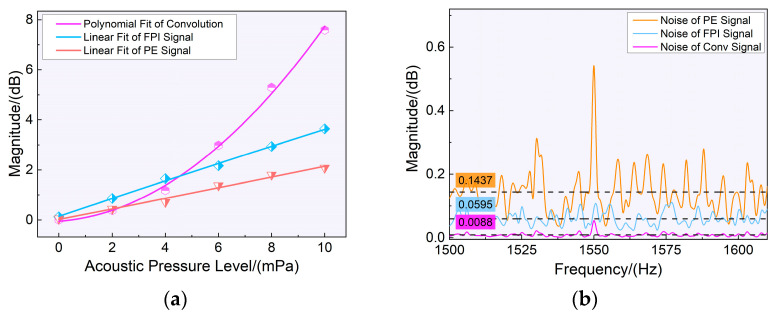
(**a**) The amplitude of the signal in the frequency domain and the amplitude after convolution at different sound pressure levels; (**b**) description of the noise level comparison.

Convolution in the time domain is multiplication in the frequency domain. In other words, if two time-domain signals are convolved, the spectrum of the resulting signal is the product of the frequency multiplications of the spectral functions of the original signals [29]. Two time-domain signal sets can consequently be filters to exclude specific elements from the other signal set. The amplitude of the same frequency component of the two signals can be raised. One-dimensional waveforms have a large number of abrupt signals and high-frequency components. In this paper, dual-channel, piezoelectric, and FPI signals were collected simultaneously, and the frequencies were the same. Therefore, convolution computation can compute the two signals to reduce the noise level and increase the signal-to-noise ratio. The time-domain signal was converted to frequency-domain processing to reduce the number of computations. According to a convolution calculation property, the signal convolution in the time domain is equivalent to multiplication in the frequency domain.

(7) $$y\left(t\right)=h(t)⊗x(t)\overset{F}{↔}Y(j\omega )\times X(j\omega ),$$

where *h(t)* and *x(t)* are the time-domain signals of the piezoelectric effect and FPI effect, respectively, and *H(jω)* and *X(jω)* are the frequency-domain signals after the Fourier transform, respectively. MATLAB is a powerful data processing and simulation software that works well with digital signals. First, the FPI and piezoelectric signals were digitized and acquired. The two groups of time-domain signals were multiplied using fast Fourier transform to improve the sensor’s ability to detect the resonance frequency and lower the signal’s noise level. The Fourier transform peak distributions of the two signals before and after convolution occurred in the appropriate locations. In Figure 7a, it is evident that below 10 mPa, the fast Fourier transform’s peak value increased by roughly three times. The convolution operation of the two signals can enhance the amplitude of the fast Fourier transform signal at the formant peak and reduce the background noise level at other frequencies. The background noise levels of the piezoelectric, FPI, and convolution signals were 0.1437, 0.0595, and 0.0088, respectively, as shown in Figure 7b. After convolution, the background noise was reduced by 85.21% compared to the FPI signal and by 93.87% compared to the piezoelectric signal. Additionally, after the convolution operation, the noise peaks for the specific frequency noises in the piezoelectric effect signals were suppressed, allowing the signal to detect acoustic signals at lower sound pressure levels and improving the sensor’s ability to resist interference.

## 4. Discussion

This paper suggests the implementation of the signal conversion of sound/light and sound/electricity using a PVDF piezoelectric film. The high piezoelectric coefficient of the PVDF film makes it ideal for use as an acoustic and deformation sensor. Additionally, the size of the diaphragm was modified to allow it to operate at a frequency of 1570 Hz in accordance with the resonance properties of the film. In the resonance state, the deformation of the film is maximized to achieve better sensitive acoustic detection. In addition, the convolution of the two signals is proposed, which significantly reduces the signal noise level and improves the signal-to-noise ratio at the resonance frequency. The frequency response output of the photoacoustic pool has apparent formant characteristics, particularly for the first-order longitudinal and Helmholtz resonant photoacoustic spectrum gas detection system. The frequency of the formant is generally within the frequency range of 1–4 kHz. In addition, this kind of photoacoustic technology is gradually maturing. In order to improve the detection ability, the dual-resonance photoacoustic spectroscopy system has gradually become a research area of great interest. The sensor established in this study is appropriate for constructing these applications. In the future, we will also apply the proposed sensor to the development and research of the photoacoustic spectrum.

## Data Availability

All data are available from the corresponding author on reasonable request.
